# A Risk Correlative Model for Sleep Disorders in Chinese Older Adults Based on Blood Micronutrient Levels: A Matched Case-Control Study

**DOI:** 10.3390/nu16193306

**Published:** 2024-09-29

**Authors:** Cheng Cheng, Xukun Chen, Liyang Zhang, Zehao Wang, Huilian Duan, Qi Wu, Ruiting Yan, Di Wang, Zhongxia Li, Ruikun He, Zhenshu Li, Yongjie Chen, Fei Ma, Yue Du, Wen Li, Guowei Huang

**Affiliations:** 1 Department of Nutrition and Food Science, School of Public Health, Tianjin Medical University, Tianjin 300070, China; chengcheng@tmu.edu.cn (C.C.); chenxk1209@tmu.edu.cn (X.C.); zly3485@tmu.edu.cn (L.Z.); wangzehao@tmu.edu.cn (Z.W.); duanhuilian@tmu.edu.cn (H.D.); wq17702280916@tmu.edu.cn (Q.W.); yanruiting1222@tmu.edu.cn (R.Y.); lizhenshu@tmu.edu.cn (Z.L.); 2 BYHEALTH Institute of Nutrition & Health, Guangzhou 510663, China; wangd7@by-health.com (D.W.); lizhongxia2013@hotmail.com (Z.L.); herk@by-health.com (R.H.); 3 Tianjin Key Laboratory of Environment, Nutrition and Public Health, Tianjin 300070, China; chenyongjie@tmu.edu.cn (Y.C.); mafei@tmu.edu.cn (F.M.); duyue@tmu.edu.cn (Y.D.); 4 Key Laboratory of Prevention and Control of Major Diseases in the Population, Ministry of Education, Tianjin Medical University, Tianjin 300070, China; 5 Department of Epidemiology and Biostatistics, School of Public Health, Tianjin Medical University, Tianjin 300070, China; 6 Department of Social Medicine and Health Management, School of Public Health, Tianjin Medical University, Tianjin 300070, China; 7 The Province and Ministry Co-Sponsored Collaborative Innovation Center for Medical Epigenetics, Tianjin 300070, China

**Keywords:** micronutrient, sleep disorders, dried blood spot technique, risk correlative model, matched case-control study

## Abstract

Background: The physical abilities of older adults decline with age, making them more susceptible to micronutrient deficiency, which may affect their sleep quality. Objectives: This study aimed to construct a risk correlative model for sleep disorders in Chinese older adults based on blood micronutrient levels. Methods: In this matched case-control study, we recruited 124 participants with sleep disorders and 124 matched controls from the Tianjin Elderly Nutrition and Cognition cohort in China. Micronutrient levels in whole blood were measured using the dried blood spot technique. We compared the differences in micronutrient levels between the two groups and also constructed a receiver operating characteristic (ROC) model and nomogram for sleep disorders. Results: In comparison to the control group, the sleep disorders group showed lower levels of blood vitamin A, vitamin E (VE), folate, magnesium, copper, iron, and selenium (Se) in the univariate analysis (*p* < 0.05). The ROC curve analysis indicated that the combination of VE + folate + Se may have an excellent diagnostic effect on sleep disorders, with an area under the curve of 0.964. This VE + folate + Se was integrated into a nomogram model to demonstrate their relationship with sleep disorders. The consistency index of the model was 0.88, suggesting that the model assessed sleep disorders well. Conclusions: The sleep disorders risk correlative model constructed by the levels of VE, folate, and Se in whole blood might show good performance in assessing the risk of sleep disorders in older adults.

## 1. Introduction

Aging is a process in which the body gradually accumulates various molecular and cellular damages, resulting in decreased function and increased risk of age-related diseases [1]. As older adults experience a decline in physical functioning with age, the incidence of sleep disorders increases [2]. The most common problem is insomnia, followed by excessive daytime sleepiness and obstructive sleep apnea [3]. In China, the overall prevalence of sleep disorders among people aged 60 years and older is 41.2% [4]. Sleep disorders can aggravate chronic non-communicable diseases in older adults and increase the social burden [5,6].

As people age, their bodies become more susceptible to micronutrient deficiencies or insufficiencies [7], affecting their sleep. A clinical trial found lower vitamin A (VA) and vitamin E (VE) levels in patients with sleep disorders compared to healthy individuals [8]. Folate also plays a crucial role in sleep quality, and supplementing with folate may positively affect sleep [9,10]. Abnormal levels of specific minerals, such as magnesium (Mg), iron (Fe), and zinc (Zn), can impact sleep quality [11], and studies have shown that supplementing with these minerals can improve sleep performance [12,13,14]. Meanwhile, a longitudinal study revealed a significant association between higher selenium (Se) intake and optimal sleep duration [15].

Previous studies focus on examining the relationship between single micronutrients and sleep quality rather than considering the combined effects of multiple micronutrients. Further research is necessary to understand better how a combination of various micronutrients can assess the sleep quality of older adults. In our study, we utilized the dried blood spot (DBS) technique to detect micronutrient levels in whole blood of older adults. Using a case-control study design, we aimed to investigate the connection between micronutrient levels and the likelihood of sleep disorders in older adults. Additionally, we aimed to create a risk-correlative model for sleep disorders to facilitate early detection and timely intervention for nutrient-deficient sleep disorders.

## 2. Materials and Methods

### 2.1. Study Design

**Figure 1:**
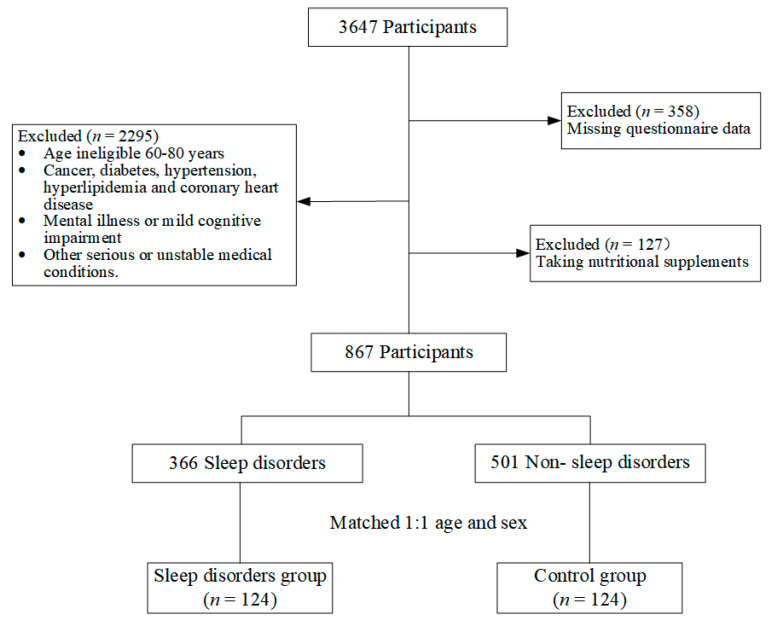
Flow chart of the study population.

All participants in this study came from the Tianjin Elderly Nutrition and Cognition (TENC) cohort (Clinical Trial Registry Identifier: ChiCTR2000034348), which included people aged 60–80 years in Tianjin. The TENC cohort had a total of 7304 individuals in 2021, of whom 3647 had whole blood samples collected. Those with missing questionnaire data and taking nutrient supplements were excluded. Exclude those persons younger than 60 or older than 80 and those with cancer, diabetes, hypertension, hyperlipidemia, coronary heart disease, mental illness, mild cognitive impairment, or other serious or unserious medical conditions. The final remaining 867 participants were screened for 366 participants with sleep disorders based on diagnostic criteria for sleep disorders, and 124 pairs of sleep disorder and control participants were matched based on propensity to match scores. Matching conditions were age (±1 year) and sex concordance, as detailed in Figure 1.

According to the sample size calculation formula for 1:1 matched case-control studies and in accordance with the relevant parameters obtained from previous literature (*OR* = 3.01, P_0_ = 0.7, α = 0.05, β = 0.1) [16,17], the minimum required sample size was 107 persons per group, and the present study included 124 persons per group, which fulfilled the sample size requirement.

Informed consent was obtained from all participants before participation. The study adhered to the principles of the Declaration of Helsinki, and the study protocol was approved by the Ethics Committee of Tianjin Medical University (Ethics No: TMUhMEC2018013) and the Ethics Committee of Baodi Clinical College of Tianjin Medical University (Ethics No: BDYYLL202404).

### 2.2. Diagnosis of Sleep Disorders

According to the Diagnostic and Statistical Manual of Mental Disorders Fourth Edition (DSM-IV) diagnostic criteria and the information collected from the questionnaires, sleep disorders were identified as having one of the following symptoms: (1) sleep onset time > 30 min, (2) excessive daytime sleepiness (ESS ≥ 11), (3) apnoea during nighttime sleep [18,19].

Sleep onset time and apnoea were self-reported by the subjects with standard questions, and excessive daytime sleepiness was assessed by scores on the Epworth Sleepiness Scale (ESS).

### 2.3. Other Variables

Training staff completed field questionnaires to gather demographic characteristics and basic information. The questionnaires collected demographic details such as age, sex, education levels, and marital status. We also gathered information on smoking, alcohol consumption, past illnesses, medication usage, and health check-ups. In addition, skilled researchers took anthropometric measurements, including height (in meters), weight (in kilograms), and waist circumference (in centimeters). The body mass index (BMI, in kg/m^2^) was then calculated based on the individual’s weight and height.

### 2.4. Measurement of Micronutrient Levels

Fasting venous blood of participants was collected into EDTA tubes by a specialized physician during physical examination and promptly transferred to the laboratory. Samples were then dispensed into centrifuge tubes and stored at −80 °C until assayed. DBS samples were made by melting the collected blood samples in a gradient and adding whole blood droplets to a DBS collection card. The DBS card was kept in a sealed foil pouch and mailed to the BYHEALTH Co., Ltd. Dried Blood Spot testing center (Guangzhou, China) for testing of vitamin (VA, vitamin D (VD), VE, vitamin B_1_ (VB_1_), vitamin B_2_ (VB_2_), vitamin B_3_ (VB_3_), vitamin B_5_ (VB_5_), vitamin B_6_ (VB_6_), folate) and mineral [Mg, copper (Cu), Fe, Zn, and Se] levels.

The samples were loaded into the rack of the DBS MS500 (CAMAG, Muttenz, Switzerland) and transferred to the internal standard spray module, and the internal standard solution was evenly sprayed onto the card. The eluate was added to the sample loop and then injected into LC-MS/MS for analysis. Vitamin levels were analyzed using an AB Sciex ExionLC system coupled to a Sciex Triple Quad 6500+ mass spectrometer.

After thawing the DBS card at room temperature, cut off the blood spots on the card with ceramic scissors and put them into centrifuge tubes, add ablution extraction solution, and sonicate the card for 40 min in Model P300H Ultrasonic Cleaner (Elma, Singen, Germany), then shake it with Model EFAA-HM-01 Vortex Mixer (ANPEL, Shanghai, China), and then centrifuge the supernatant with Model 3K15 High-Speed Centrifuge (SIGMA, Rodelmark, Germany), and then the supernatant was taken for measurement. Mineral levels were detected using an inductively coupled plasma mass spectrometer (Agilent 7800 ICP-MS, Santa Clara, CA, USA).

### 2.5. Statistical Analysis

Cumulative frequency distributions of continuous variables were tested using the Kolmogorov–Smirnov normality test, the results of which were expressed as mean ± standard deviation (SD), and categorical variables were expressed as numbers (percentages). Demographic characteristics and basic information differences between the case and control groups were compared using the Student’s *t*-test or Wilcoxon rank-sum test for continuous variables and χ^2^ test for categorical variables based on the data distribution characteristics.

The study used a conditional logistic regression model to investigate the link between multiple micronutrient levels and sleep disorders. The model calculated odds ratios (*OR*) and 95% confidence intervals (95%CI) to assess the risk of sleep disorders. The regression model categorized mineral concentrations as either low or high based on the median value. Model 2 accounted for educational attainment, smoking status, drinking status, BMI, and waist circumference, while Model 1 was a univariate model.

We used random forest (RF) modeling to demonstrate how vitamin and mineral levels impact sleep disorders. RF is a type of ensemble learning that uses a decision tree as the primary classifier. It involved randomly selecting attributes during training and combining the results from multiple decision trees to make the final classification [20]. The study used receiver operating characteristic (ROC) curve analysis to evaluate the role of micronutrients important in the risk of developing sleep disorders after RF screening. The differences in the area under the curve (AUC) for each combination of micronutrients were compared. A nomogram model was used to score the risk factors in the ROC model to assess sleep disorder risk. The consistency index (C-index) was utilized to determine the discrimination and calibration of the assessment models.

Statistical analyses were performed using R software (4.1 Version 0, R Foundation for Statistical Computing) and SPSS 24.0 (IBM, Chicago, IL, USA). All statistical tests were two-sided, and differences were considered statistically significant at a *p*-value of less than 0.05.

## 3. Results

### 3.1. Demographic and Clinical Characteristics of the Study Population

This research included 124 patients with sleep disorders and 124 matched healthy controls. The mean age was 69.1 ± 4.3 years, with 146 (58.9%) females. Table 1 shows the baseline characteristics of the study participants. We compared the basic characteristics of the total sleep disorder population remaining after the exclusion of disease and the matched sleep disorder population included in this study, and the results showed that the differences between the basic characteristics of the total sleep disorder population and the matched sleep disorder population were not statistically significant (*p*^1^ > 0.05). Therefore, the population we included can represent the population with sleep disorders in this cohort. There was no statistically significant difference between the matched sleep disorders and control group regarding education levels, smoking status, alcohol consumption status, marital status, BMI, and waist circumference (*p*^2^ > 0.05).

### 3.2. Comparison of the Micronutrient Levels between the Sleep Disorders and Controls

The results of comparing the vitamin and mineral content of the study population in the sleep disorders and control groups are shown in Table 2. Compared to the control group, the sleep disorders group exhibited lower levels of VA, VE, folate, Mg, Cu, Fe, and Se, and these differences were statistically significant (*p* < 0.05).

### 3.3. Multiple Micronutrient Status and Risk of Sleep Disorders

To investigate the relationship between the combined effect of the levels of the seven micronutrients mentioned above and sleep disorders, we categorized the levels of the seven micronutrients into two groups, high and low, based on the median micronutrient concentration in whole blood. We defined poor micronutrient states as having more than three micronutrients lower than average. After adjusting for educational levels, smoking status, alcohol drinking status, BMI, and waist circumference, the results showed that older adults with poor micronutrient states might have a higher risk of sleep disorders (*OR*: 8.27, 95%CI: 3.89–17.54, *p* < 0.001). The results of comparing the relationship between multiple micronutrient levels and sleep disorders are presented in Table S1 and Figure S1 of Supplementary Material.

### 3.4. RF of Micronutrients on Sleep Disorders Impacts

**Figure 2:**
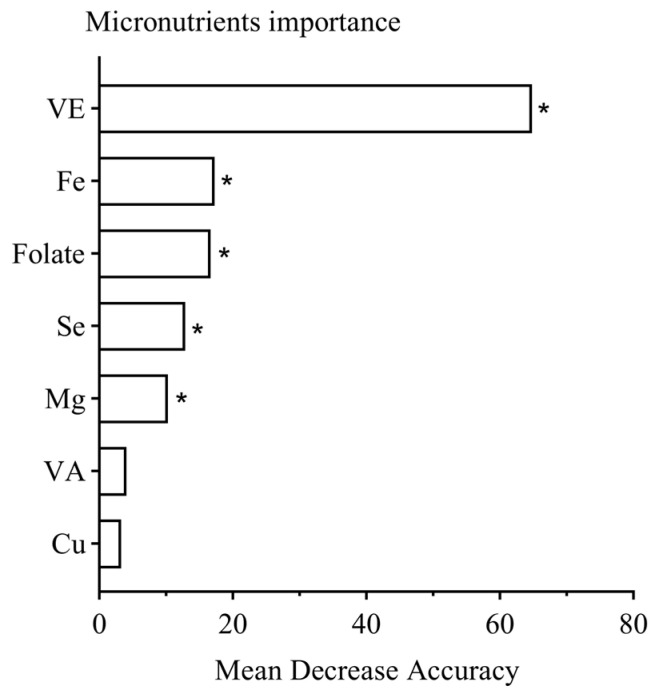
The importance of micronutrients for sleep disorders. *, *p* < 0.05.

The micronutrients that showed statistical differences between the two groups were used in the RF model. The more the mean accuracy of the metrics in the RF model decreases, the more important the feature is to the model. The results of the ranking of the importance of vitamins and minerals for sleep disorders are presented in Figure 2. Among these, VE was found to be the most significant nutrient in terms of its effects on sleep disorders, followed by Fe, folate, Se, and Mg in that order (*p* < 0.05), while VA and Cu were relatively less important regarding its impact on sleep disorders (*p* > 0.05).

### 3.5. ROC Analysis of Micronutrients with Sleep Disorders

**Figure 3:**
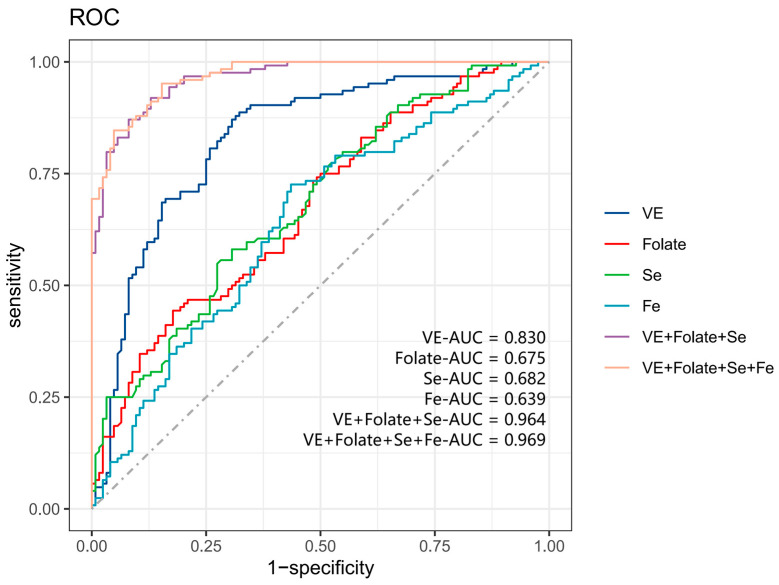
ROC for assessing sleep disorders. ROC, receiver operating characteristic; AUC, area under the curve.

We selected micronutrients with relatively high single importance in the RF model and put them into the ROC model to establish a risk-correlative model for sleep disorders. The diagnostic effects of VE, folate, Fe, and Se alone and in combination with sleep disorders are shown in Figure 3. The AUC of VE, folate, Se, and Fe were 0.830, 0.675, 0.682, and 0.639, respectively. The AUC of VE + folate + Se in combination and VE + folate + Fe + Se in combination were 0.964 and 0.969. The diagnostic effects of the individual micronutrients alone were smaller than those of the combination of VE + folate + Se for the diagnosis of sleep disorders.

In order to find the optimal combination to establish a correlative model for sleep disorders, we compared the differences in diagnostic performance between the micronutrient combinations, and the results are illustrated in Figure S2 of Supplementary Material. The difference in AUC between the ROC models established with the VE + folate + Fe + Se + Mg combination and the VE + folate + Fe + Se combination was not statistically significant (*p* > 0.05). In comparison to the combination of VE + folate + Fe + Se, the AUC was lower for both VE + folate +Fe and VE + Fe + Se (*p* < 0.05). However, the difference between the combination of VE + folate + Se and the combination of VE + folate + Fe + Se was not statistically significant (*p* > 0.05). Furthermore, compared to the combination of VE + folate + Se, the AUC was lower for both the combination of VE + folate, VE + Fe, and VE + Se (*p* < 0.05), and these findings are presented in Figure S3 of Supplementary Material. As a result, we decided to devise a risk correlative model for sleep disorders using a combination of VE + folate + Se.

We conducted another validation study to evaluate the reliability of the ROC results. In Figure S4 of Supplementary Material, we use the randomized split validation method, which splits the data into a training set and a validation set in a 7:3 ratio to internal validation of the ROC model. The results demonstrated that the difference in the AUC between the training and validation models was not statistically significant (*p* > 0.05), indicating the effectiveness of using three micronutrients to diagnose sleep disorders.

### 3.6. Nomogram Analysis of Micronutrients with Sleep Disorders

**Figure 4:**
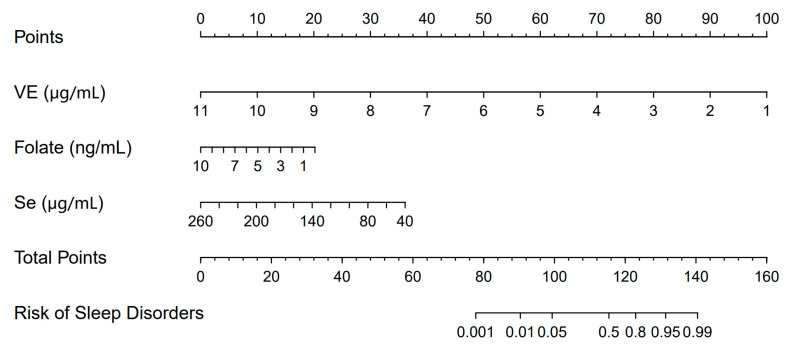
Nomogram to assess the probability of sleep disorder events in participants. C index = 0.880.

Our current research combined VE, folate, and Se into a Nomogram model. Then, we displayed it in a Nomogram to illustrate the connection between these three micronutrients and sleep disorders in whole blood, as presented in Figure 4. Each nutrient’s scale represents its range of values, and the line length indicates the extent of its impact on sleep disorders. VE made the most significant contribution, followed by Se and folate. The model’s C-index was 0.88, indicating that it effectively evaluates sleep disorders.

## 4. Discussion

This study examined the relationship between micronutrient levels in whole blood and the risk of sleep disorders using a matched case-control design. We also created a model to gauge the risk of sleep disorders based on those micronutrient levels. The findings indicate that there may be an association between levels of VA, VE, folate, Mg, Cu, Fe, and Se in whole blood and the risk of sleep disorders. We defined poor micronutrient states as having more than three micronutrients below the average. Older adults with poor micronutrient states may exhibit a higher risk of sleep disorders (*OR*: 8.27, 95%CI: 3.89–17.54, *p* < 0.001). Furthermore, by incorporating these micronutrients in the RF model, we found that VE, folate, Mg, Fe, and Se levels may have some impact on sleep disorders. The ROC model established by combining VE, folate, and Se may have a better effect on the diagnosis of sleep disorders among those micronutrients. Moreover, constructing a nomogram model using these micronutrients further provided a dependable assessment of sleep disorders.

A significant amount of research focuses on micronutrients and sleep, providing evidence that micronutrient deficiencies can affect sleep [21]. An observational cross-sectional study found that patients with obstructive sleep apnoea, with an average age of 36, had lower levels of VE compared to the general population [22]. A one-month randomized placebo-controlled trial in Thailand showed that VE supplementation significantly improved sleep quality in postmenopausal women with chronic insomnia [23]. Folate, which carries one carbon unit, participates in various metabolic reactions in the human body and can impact human sleep [24]. A study involving 24,234 participants from the United States reported that B vitamins, including folate, may have a protective effect on sleep [25,26]. A prospective cohort study conducted at Peking University, which included 4352 women, suggested that pre-pregnancy folate intake might be a protective factor against decreased sleep quality in pregnant women [27]. Researchers generally support the association of minerals with sleep, particularly the beneficial effects of Se, Fe, and Mg. A cross-sectional study including 3660 participants showed that higher dietary Se intake can help adults achieve optimal or longer sleep [11]. Similarly, a case-control study from Taiwan, China, involving 64 participants with an average age of 40–45 years found that patients with obstructive sleep apnea had low levels of erythrocyte Se and that their apnea/hypopnea index was also negatively correlated with erythrocyte Se concentration [28]. Another study involving 38 pregnant women with restless legs syndrome showed that reduced serum Se levels were significantly associated with impaired sleep quality in patients with restless legs syndrome [29]. Other minerals, such as Fe and Mg levels, are positively correlated with sleep duration, and dietary supplements like Fe and Mg have been reported to improve sleep significantly [30,31]. However, there is a lack of research on the relationship between multiple micronutrients and sleep function, and there is no direct evidence from the Chinese elderly population.

Risk prediction models for sleep disorders typically use BMI, sex, neck circumference, snoring, and blood pressure as predictors [32]. Some models also incorporate reversible predictors such as C-reactive protein, albumin, calcium, and parathyroid hormone to forecast the risk of sleep disorders [33]. Additionally, some models utilize simple questionnaires and machine-learning techniques for prediction [34]. However, the predictor detection process in these prediction models is complex and time-consuming, and the questionnaires are prone to information bias, making practical application difficult. To address the limitations of the original prediction model, we developed a data model to assess sleep disorders in elderly Chinese individuals using multiple micronutrients. It is important to note that the DBS technique used in this study can rapidly and accurately detect levels of various micronutrients in whole blood and assess the risk of sleep disorders based on the established assessment model.

This study established a correlative model for sleep disorders in older adults using whole blood levels of VE, folate, and Se as factors. Although Mg and Fe were also found to affect sleep, the results showed that Mg and Fe were not as important as other micronutrients and were excluded from the model. After comparing different models, it was found that the model that only included VE, folate, and Se had the best diagnostic effect for sleep disorders. As a result, a nomogram model based on these three micronutrients was established to assess the risk of sleep disorders in older adults.

This study has the following strengths. First, most existing studies focus on the relationship between a single nutrient and sleep disorders. Since the association between a single micronutrient and sleep status is not strong, there is a need for data models that combine multiple micronutrients to assess sleep status. Second, we applied the DBS technique to detect micronutrient levels in whole blood. DBS technique combines DBS sample extraction, processing, and micronutrient level detection, which allows for automated sample detection [35,36] and offers simple sampling, lower transportation and storage costs, and quick and convenient processing [37,38,39]. Finally, our evaluation model incorporates objective indicators less influenced by subjective factors, making the resulting model more reliable.

There are some limitations in this study. First, it was an observational study and could not confirm the causal relationship between micronutrients and sleep disorders, which needs to be explored in further longitudinal studies. Second, although some internal validation has been conducted to assess the reliability of the ROC results, external validation is still required. Finally, the sample size of this study is small, and it is a single-center study. Therefore, it is necessary to conduct more large-scale research.

## 5. Conclusions

In summary, there may be a correlation between the levels of seven nutrients (VA, VE, folate, Fe, Mg, Cu, and Se) in whole blood and the risk of sleep disorders. It was observed that the higher the number of nutrients at lower levels, the greater the risk of sleep disorders. Additionally, based on the levels of VE, folate, and Se in whole blood, the nomogram model performed well in personalized assessment of the risk of sleep disorders in older adults. This model may assist medical researchers in conducting early screening for sleep disorders and developing relevant prevention strategies.

## Figures and Tables

**Table 1:** Comparison of key characteristics of the study participants.

Characteristics	Total Sleep Disorders (*n* = 366)	Matched Sleep Disorders (*n* = 124)	Control (*n* = 124)	*p*^1^ Value	*p*^2^ Value
Age (years)	68.60 ± 4.52	69.1 ± 4.3	69.1 ± 4.3	0.400	1.000
Sex, male (%)	135 (36.90)	51 (41.1)	51 (41.1)	0.284	1.000
Education levels, *n* (%)				0.144	0.157
Illiteracy	241 (65.8)	61 (49.2)	46 (37.1)		
Primary school	88 (24.0)	29 (23.4)	36 (29.0)		
Secondary school and above	37 (10.1)	34 (27.4)	42 (33.9)		
Smoking status, *n* (%)				0.748	0.221
Non-smoker	239 (65.3)	79 (63.7)	89 (71.8)		
Ex-smoker and smoker	127 (34.7)	45 (36.3)	35 (28.2)		
Alcohol drinking, *n* (%)				0.952	0.353
Yes	299 (78.1)	23 (18.5)	30 (24.2)		
No	67 (21.9)	101 (81.5)	94 (75.8)		
Marital status, *n* (%)				0.476	0.683
Married or cohabited	330 (90.2)	109 (87.9)	112 (90.3)		
Single, separated, or widowed	36 (9.8)	15 (12.1)	12 (9.7)		
BMI (kg/m^2^)	25.18 ± 3.17	25.61 ± 3.66	25.96 ± 3.56	0.21	0.433
Waist circumference (cm)	87.82 ± 9.02	88.99 ± 10.07	90.36 ± 8.38	0.224	0.252

BMI, body mass index; Paired *t*-test, mean ± SD; χ^2^ test, n (%). *p*^1^ represents the *p*-value of the total sleep disorder population compared to the basic characteristics of the matched sleep disorder population included in this study. *p*^2^ represents the *p*-value for the comparison of the basic characteristics of the matched sleep disorder group with those of the control group.

**Table 2:** Vitamin and mineral levels of the study population.

Micronutrients	Sleep Disorders (*n* = 124)	Control (*n* = 124)	*p* Value
VA (ng/mL)	310.13 ± 128.85	366.74 ± 140.32	<0.001
VD (ng/mL)	35.70 ± 13.70	36.90 ± 12.90	0.482
VE (μg/mL)	2.56 ± 0.59	3.61 ± 1.17	<0.001
VB_1_ (ng/mL)	0.86 ± 0.66	0.82 ± 0.48	0.774
VB_2_ (ng/mL)	3.65 ± 1.75	4.03 ± 3.32	0.942
VB_3_ (μg/mL)	8.64 ± 2.34	8.90 ± 2.21	0.442
VB_5_ (μg/mL)	47.50 ± 22.74	54.15 ± 50.64	0.995
VB_6_ (ng/mL)	9.74 ± 6.43	10.60 ± 6.29	0.181
Folate (ng/mL)	4.51 ± 2.35	6.02 ± 2.24	<0.001
Mg (μg/mL)	36.99 ± 7.59	39.63 ± 6.11	0.002
Cu (ng/mL)	794.14 ± 155.56	966.34 ± 690.63	0.002
Fe (μg/mL)	362.83 ± 77.36	401.37 ± 89.24	<0.001
Zn (μg/mL)	7.51 ± 2.60	8.88 ± 7.62	0.294
Se (ng/mL)	95.70 ± 21.83	113.03 ± 26.30	<0.001

All variables were not normally distributed and are expressed as mean ± SD for presentation purposes. Wilcoxon Signed Rank Test was used to compare the variation in micronutrient levels between the two groups.

## Data Availability

The raw data supporting the conclusions of this article will be available upon request from the corresponding author.

## Supplementary Materials

The following supporting information can be downloaded at https://www.mdpi.com/article/10.3390/nu16193306/s1, Figure S1: The conditional logistic regression on micronutrient statues related to sleep disorders; Figure S2. ROC for predicting sleep disorders based on different combinations; Figure S3. ROC for predicting sleep disorders based on different combinations; Figure S4. ROC for comparison of validation and training models; Table S1: Different micronutrients level groups of the study population.
