# Supplementary material for: A Risk Correlative Model for Sleep Disorders in Chinese Older Adults Based on Blood Micronutrient Levels: A Matched Case-Control Study
Source: Nutrients. 2024 Sep 29;16(19):3306. doi: 10.3390/nu16193306 (PMC11479059; doi:10.3390/nu16193306)
Supplement: Supplementary file 1 [file nutrients-16-03306-s001.zip › nutrients-3222481-supplementary.pdf]

Supplementary material

Table S1. Different micronutrients level groups of the study population

Micronutrient states	Sleep disorders (*n*=124)	Control (n=124)	*p* value
Group			<0.001
High	39 (31.5%)	89 (71.8%)	
Low	85 (68.5%)	35 (28.2%)	

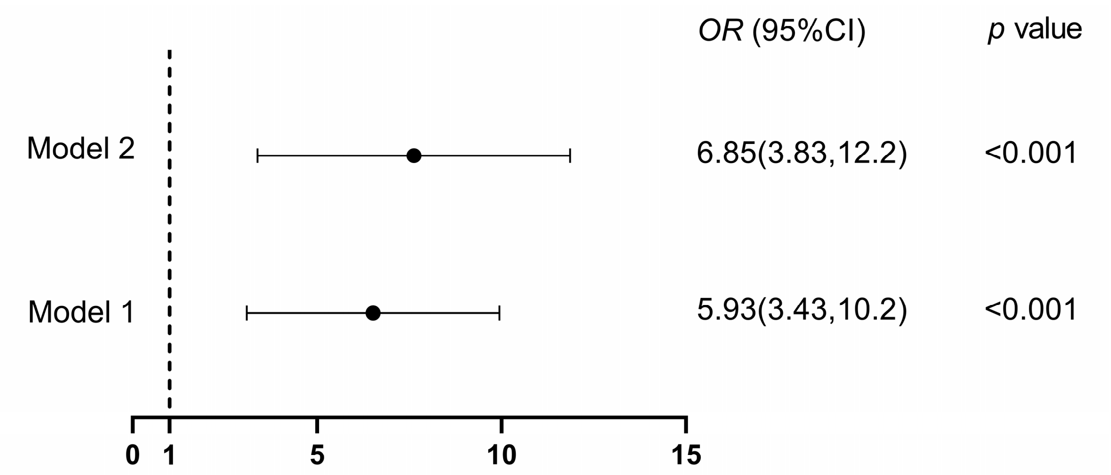

Figure S1. The conditional logistic regression on micronutrient statues related to sleep disorders. Model 1 is non-adjusted model. Model 2 is adjusted for education levels, smoking status, alcohol drinking, BMI and waist circumference. OR, odds ratios; 95% CI, 95 % confidence intervals.

In order to find the optimal combination to establish the prediction model, we compared the differences in prediction performance between the micronutrient combinations and the results are illustrated in Figure S2 of supplementary material. The difference in AUC between the ROC models established with the VE + folate + Fe + Se + Mg combination and the VE + folate + Fe + Se combination was not statistically significant ($p > 0.05$). Compared to the combination of VE + folate + Fe + Se, the AUC was lower for both the combination of VE + folate +Fe and the combination of VE + Fe + Se ($p < 0.05$), and the results are shown in Figures S2 of supplementary material. However, the difference between the combination of VE + folate + Se and the

combination of VE + folate + Fe + Se was not statistically significant ($p > 0.05$). Therefore, we chose to establish a risk prediction model for sleep disorders with a combination of VE + folate + Se.

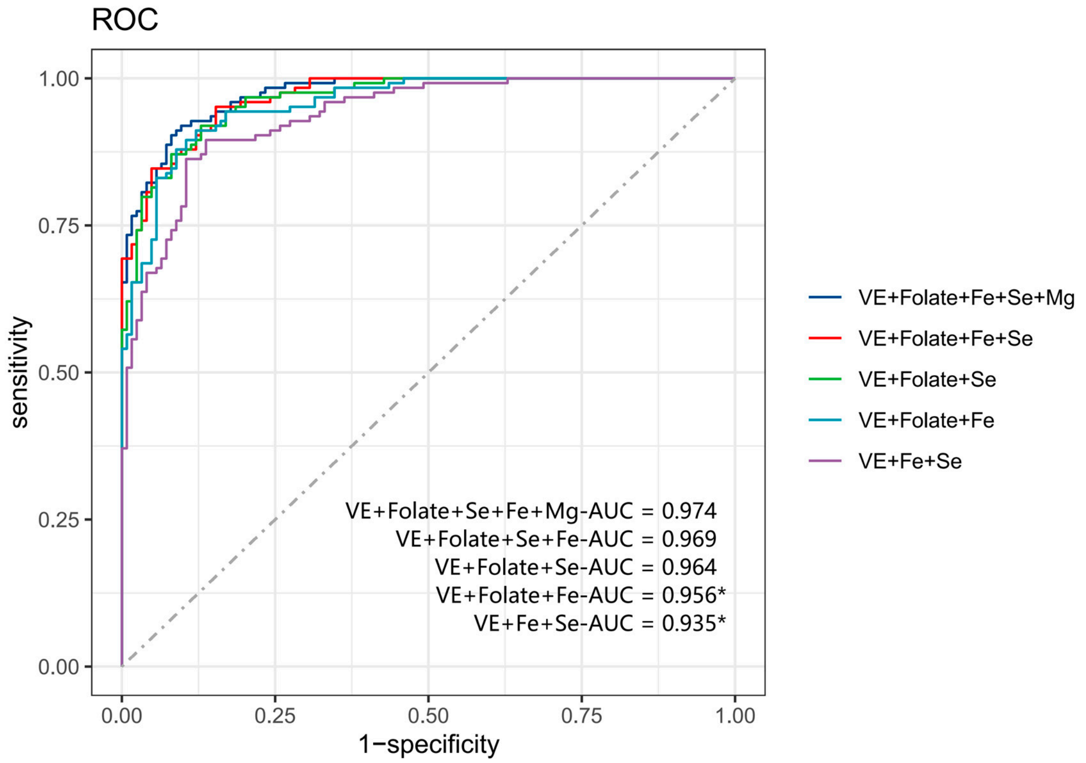

Figure S2. ROC for predicting sleep disorders based on different combinations. ROC, receiver operating characteristic; AUC, area under the curve. * The difference was statistically significant when compared to VE ± folate ± Se ± Fe.

Meanwhile, compared to the combination of VE + folate + Se, the AUC was lower for the combination of VE + folate, VE + Fe and VE + Se ($p < 0.05$). Therefore, the prediction model with the combination of VE + folate + Se was the best for sleep disorders assessment.

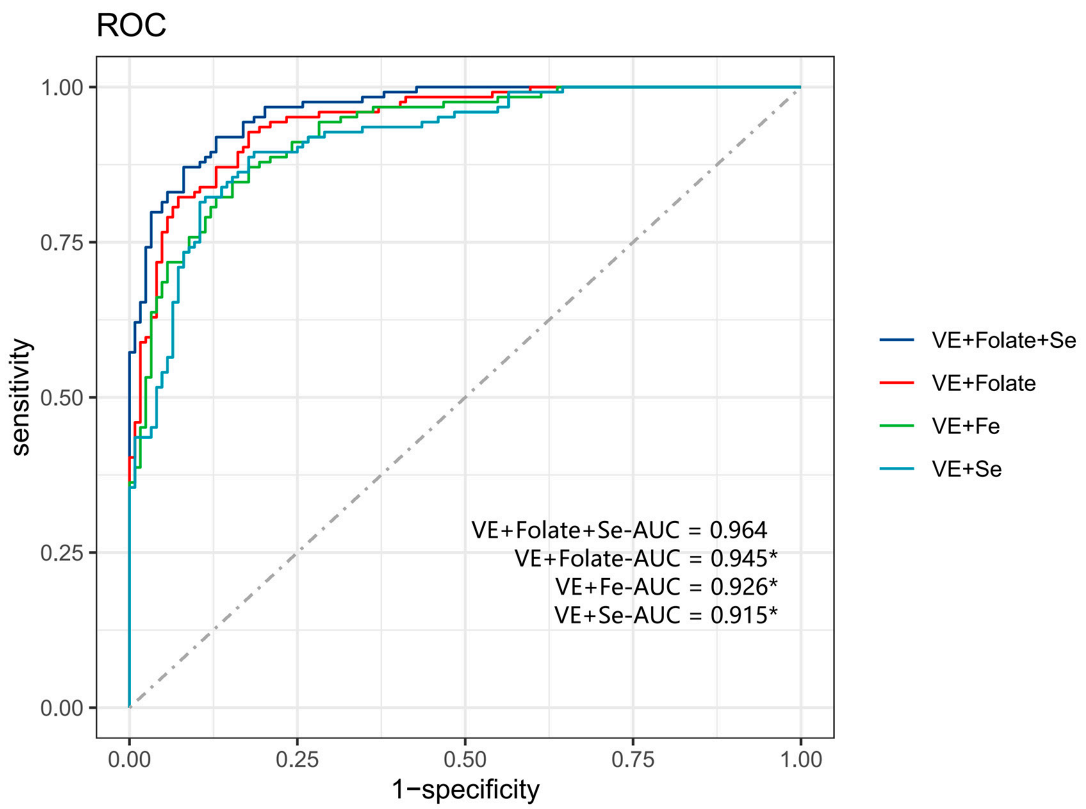

Figure S3. ROC for predicting sleep disorders based on different combinations. ROC, receiver operating characteristic; AUC, area under the curve. * The difference was statistically significant when compared to VE ± folate ± Se.

Internal validation of the ROC model was performed using the random split validation method, where the data were randomly split 7:3 to form the training and validation sets. There was no significant difference in the AUC of VE, Se, folate and combination, and the model was considered to have good accuracy.

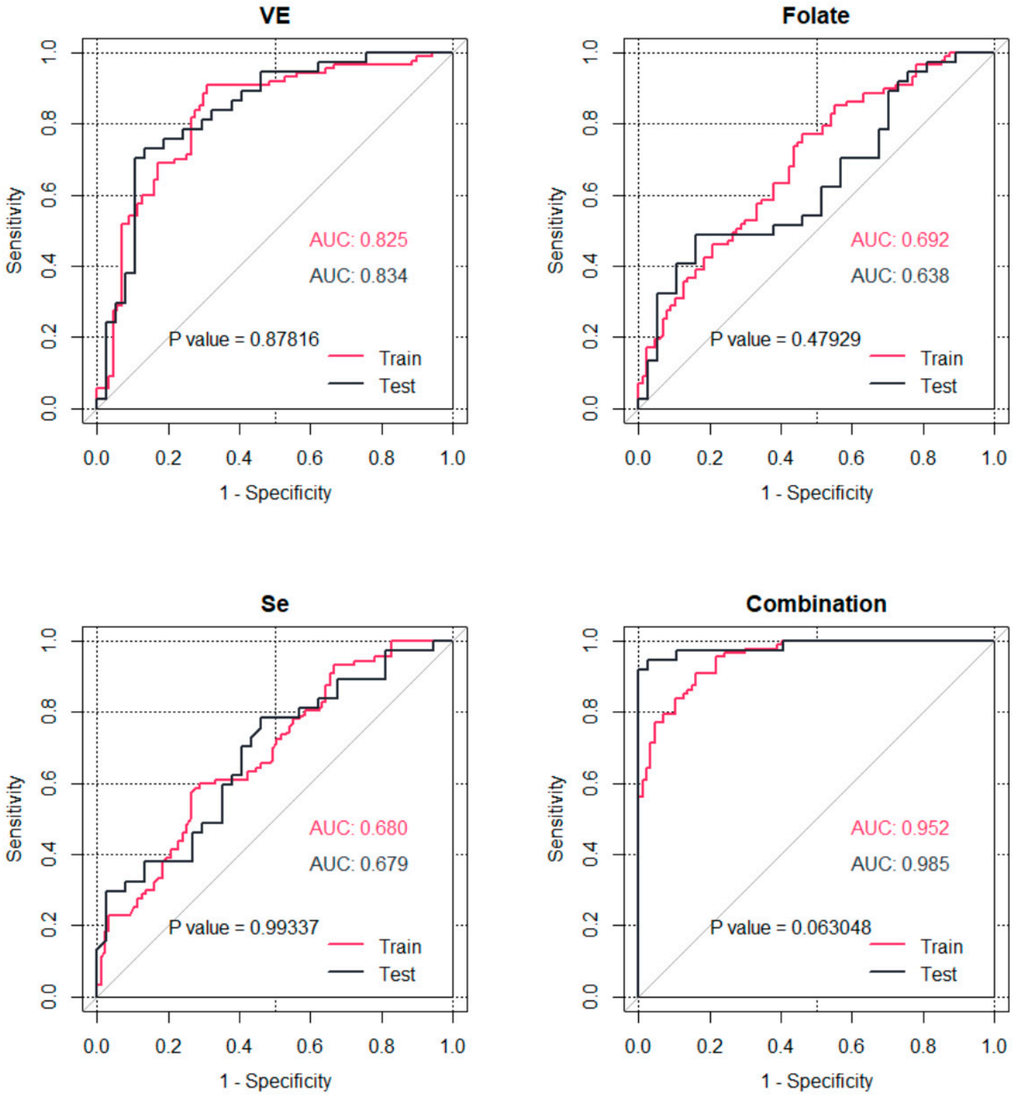

Figure S4. ROC for comparison of validation and training models. ROC, receiver operating characteristic; AUC, area under the curve.
